# Predicting ecosystem metaphenome from community metagenome: A grand challenge for environmental biology

**DOI:** 10.1002/ece3.9872

**Published:** 2023-03-08

**Authors:** Neo D. Martinez

**Affiliations:** ^1^ Center for Complex Networks and Systems, School of Informatics, Computing, and Engineering Indiana University, Bloomington Indiana Bloomington USA; ^2^ Pacific Ecoinformatics and Computational Ecology Lab CA Berkeley USA

**Keywords:** computation, data science, ecology, ecosystem, environmental nucleic acids, evolution, networks, prediction, synthesis, theory

## Abstract

Elucidating how an organism's characteristics emerge from its DNA sequence has been one of the great triumphs of biology. This triumph has cumulated in sophisticated computational models that successfully predict how an organism's detailed phenotype emerges from its specific genotype. Inspired by that effort's vision and empowered by its methodologies, a grand challenge is described here that aims to predict the biotic characteristics of an ecosystem, its metaphenome, from nucleic acid sequences of all the species in its community, its metagenome. Meeting this challenge would integrate rapidly advancing abilities of environmental nucleic acids (eDNA and eRNA) to identify organisms, their ecological interactions, and their evolutionary relationships with advances in mechanistic models of complex ecosystems. Addressing the challenge would help integrate ecology and evolutionary biology into a more unified and successfully predictive science that can better help describe and manage ecosystems and the services they provide to humanity.

## Graphical abstract

A grand challenge is described that attempts to predict the structure, function, and dynamics of an ecosystem, its metaphenome, from the nucleic acid sequences of organisms within the ecosystem's communities, its metagenome. Meeting the challenge integrates rapidly advancing technologies that enable environmental DNA and RNA to identify organisms and their interactions with advances in mechanistic modeling of complex ecosystems. The challenge aims to leverage the vision and methodologies of an analogous challenge in systems biology to predict organismal phenotype from its genotype. Meeting the challenge may advance and synthesize environmental biology so that it more effectively contributes to addressing key threats to biodiversity and ecosystem services.

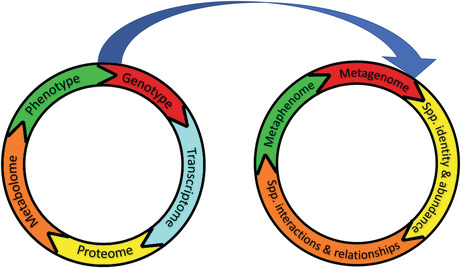

## 1 INTRODUCTION

**FIGURE 1:**
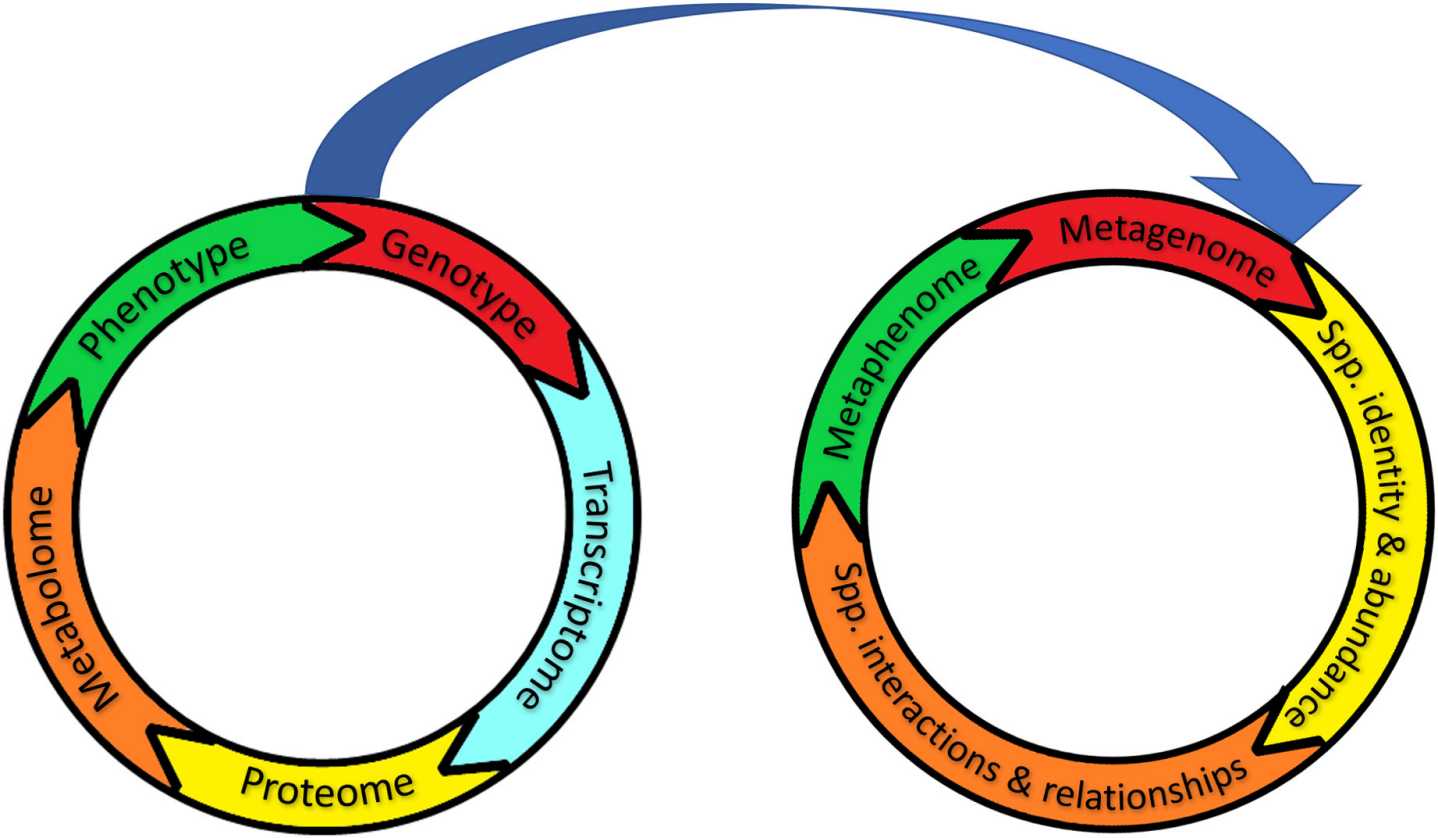
Simplified depiction (after Bathe & Farshidfar, 2014) of how an organism's phenotype *emerges* from its genotype (left) and its role in an analogous depiction (right) of how an ecosystem's biotic characteristics, its metaphenome, may be *predicted* from the genomes of its constituent species, its metagenome. The ability to identify species and quantify their abundance stems from (upper blue arrow) the ability of genotypes to identify the phenotype (e.g., body type and size, taxonomy, etc.) of organisms. Similar colors between left and right depictions indicate analogous steps of emergence. While proteomes are biologically derived from genotypes via the transcriptome, the identity and abundance of species may be bioinformatically derived from an ecosystem's metagenome. This enables the identification of species' ecological interactions that function similarly to the metabolome in generating essential characteristics of organisms and ecosystems. For example, a key similarity is that both phenotypes and metaphenomes (green) emerge from networks of interactions (orange) among functionally similar molecules (left) and organisms (right) grouped into functionally distinct species (yellow) that interact at concentration‐ (left) and density‐ (right) dependent rates modeled by Michaelis–Menten functions (left) also called “functional responses” by ecologists (right, Fang et al., 2020; Martinez, 2020).

“Grand Challenges” have emerged as one of the most compelling tools to motivate, engage, and organize major research programs across the sciences and engineering (Kaldewey, 2018; Omenn, 2006). This is especially true for biology. Grand challenges in evolution include assembling a great tree of life summarizing the evolution of all life on our planet (Hinchliff et al., 2015). Grand challenges in ecology include understanding the relationship between biodiversity and ecosystem function (National Research Council, 2001; van der Plas, 2019). One of the largest and most successful grand challenges since sequencing the human genome includes molecular and cell biologists' project to predict individual organisms' characteristics from their DNA sequence otherwise known as predicting an organism's phenotype from its genotype (Figure 1, National Research Council, 2010). This challenge was largely met for one species by a whole‐cell simulation of a human pathogen (Karr et al., 2012) as proposed a decade earlier (Tomita, 2001).

Mechanistic models of phenotypes emerging from their genotypes continue to be developed for different organisms using a variety of approaches. Several approaches employ empirically parameterized models of complex networks comprised of modules using mathematical and other algorithms to represent DNA transcription, translation of RNA into proteins, and metabolic processes involving those proteins (Figure 1, Fang et al., 2020; Karr et al., 2012). Others model individual molecules and the cell's physical structure created by those molecules to generate much more highly resolved representations of cells including membranes, organelles, proteins, and their interactions (Feig & Sugita, 2019). Both approaches include many types of interactions involving thousands of biochemical species and parameters operating on multiple scales from molecular dynamics to cell division. The sophistication and coordinated research and engineering applied to modeling whole cells vastly exceeds that dedicated to similarly detailed modeling of whole ecosystems. Both inspired and informed by whole‐cell modeling as well as a long history of more modest behavioral, population, community and ecosystem theory and modeling, the grand challenge to predict ecosystem metaphenome from community metagenome seeks to motivate a multiscale mechanistic understanding of how the detailed structure and function of ecosystems emerge from the interactions among organisms coexisting within an environment (Jansson & Hofmockel, 2018).

Addressing this challenge helps integrate behavioral, population, community, and ecosystem ecology, several of the largest subdisciplines of ecology, with phylogenetics and population genetics, two of the largest subdisciplines of evolutionary biology. Together, ecology and evolution comprise much of environmental biology which has yet to achieve the synthetic and predictive successes enjoyed by physics, chemistry, and molecular and cell biology. Such limitations may be surmounted by predicting ecosystem metaphenomes from the metagenomes within the ecosystem's communities starting first with relatively simple experimental ecosystems in the laboratory and eventually extending to ecosystems more generally. Ecological communities are described by the diversity and identity of species within a habitat and are identifiable by their genetic “barcodes.” The community metagenome consists of the genomes of all species within the ecosystem's communities. A key metaphenome is the distribution of organisms among all trophic levels within a habitat and the dynamics of their populations and biomass over time. Cell and molecular biologists' success at meeting their genotype‐to‐phenotype challenge points toward the tractability of a similar challenge at the ecosystem level and contributes powerful social (e.g., structured collaborations), scientific (e.g., networks of networks), and technical (e.g., sequencers and software) methodologies for addressing the challenge. What the grand challenge is, how it may be met, and why it is worth pursuing are described further below.

## 2 THE CHALLENGE

To scale up our understanding of organismal behavior and interactions to the structure and function of ecosystems, it is eminently clear that knowing which organisms and interactions occur in an ecosystem is a useful place to start. Documenting these ecosystem traits has been a priority since well before Darwin's voyages. More recently, rapidly emerging technologies based on environmental nucleic acids (eNA) including eDNA and eRNA have greatly increased our ability to identify these traits at a vastly higher degree of resolution with extraordinarily lower effort and cost (Beng & Corlett, 2020; Deiner et al., 2021). These technologies illuminate the community metagenome constituted by the genomes within ecosystems which provides a uniquely powerful description of the current state and dynamic potential of the ecosystem (Deiner et al., 2021; Jansson & Hofmockel, 2018). Sequences of eDNA within metagenomes may identify organisms at virtually any level of taxonomic resolution from the species level and above down through the population level (Luck et al., 2003) to the individual organism itself including phylogenetic information about organisms' evolutionary history. Thus, metagenomes elucidate who organisms are, where they come from, their evolutionary potential, and help leverage existing data on well‐known organisms (e.g., body size, diet, etc.) that often accurately describe less familiar close relatives (Davies, 2021). The location of metagenomes such as within the tissue, gut, or pollen sacks of an organism, combined with the organism's known biology such as whether it's an animal, plant, parasite, pollinator, etc., can illuminate interactions such as predation, parasitism, herbivory, symbiosis, and mutualism (Kennedy et al., 2020). Innovative methods, such as separating differently sized DNA molecules, can distinguish endosymbionts and parasites from prey (Krehenwinkel et al., 2017). The number of eNA copies can even elucidate species' biomass, population size, life‐stage structure, eco‐evolutionary dynamics (Hao et al., 2020), organismal interactions (e.g., feeding, pollination, and growth, Deiner et al., 2021; Yates et al., 2021) and rates of ecosystem processes (Kennedy et al., 2020).

By rigorously linking to the vast literature on the physiology, natural history, and ecology of identified organisms, the metagenome can unlock much if not most of the empirical information essential to predicting ecosystem behavior including virtually all co‐existing organisms' identities and their most important and cryptic interactions. Correlations between species composition and habitat may allow inferences such as the size, location, and hydrologic regime of a lake or the climate, soil type, and fertility of a grassland. As such, ecoinformatic analyses of barcodes within the metagenome (as opposed to mechanistic simulations of phenotypes emerging from genotypes) including intraspecific variation among populations may elucidate the biotic and abiotic structure of the ecosystem needed to be modeled and ecosystem dynamics to be simulated. Such analyses can be supplemented with additional data including environmental observations and more mechanistic genotype‐to‐phenotype inferences, especially among microbes, such as the presence of nitrogenase eRNA indicating nitrogen fixation. The subsequently identified organisms, traits, and dynamics form much of the ecosystem's metaphenome to be predicted from, and test, models that formalize theories of how community metagenomes may enable scientists to predict ecosystem metaphenomes (Table 1).

**TABLE 1:** Grand challenge steps and key limitations (Gilbert & Lynch, 2019) for generally predicting ecosystem metaphenomes from community metagenomes compared to specifically predicting Lake Constance's metaphenome (Boit et al., 2012) with Allometric Trophic Network (ATN) theory (Martinez, 2020).

Step	Challenge	Focus	Current limitations	Allometric trophic network theory
1	Metagenotyping	Habitats, organisms, guts, tissues	Spatiotemporal and phylogenetic sampling resolution	Simulations based on inputs from 30 yr of observing L. Constance biota
2	Identify and quantify	Taxa, diversity, phylogenies, populations, growth rates, interactions	Barcode quality/correlation w/traits, database integration, and coverage	Field and laboratory observations & experiments
3	Assemble and parameterize consumer‐resource networks	Feeding, infection, pollination, seed dispersal, nutrient transport, habitat provision, decomposition, preferences, adaptive responses (fear, cooperation, interference, defense, etc.)	Creating, testing, and measuring parameters of improved functional responses, including more effects of environmental, ecological, and temporal variability on interactions	25 Nodes: detritus, decomposers, bacteria, phytoplankton, zooplankton, fishes. Empirically observed & allometrically estimated parameters.
4	Code and conduct simulations	Uncertainty, variability, stability, species abundance & distribution. Effects of species loss & invasion, warming & eutrophication, extraction	Determining which ecosystem traits can be confidently predicted including sensitivity of predictions to context and model uncertainty	Seasonal dynamics of a complex food web with 1‐day time steps initialized with spring conditions
5	Test against observations	Self‐consistency, controlled & uncontrolled experiments	Achieving success in laboratory conditions and scaling success into the field, collaboration on model ecosystems	Explains 83% & 88% seasonal bio‐mass & production variability of 20 auto‐ & heterotrophic spp.+ detritus
6	Repeat 1–5 as necessary	Test novel predictions, explore new hypotheses	Demonstrating scientific advances warrant research effort and costs	Address age‐structure, fishing, environmental noise & mutualism

*Note*: ATN predictions of ecosystem metaphenomes involve simulations of empirical and niche‐model networks of consumer–resource interactions that have been broadly corroborated empirically (Martinez, 2020): Vucic‐Pestic et al. (2011) predicted the recently observed decreases in trophic transfer efficiency (Barneche et al., 2021) caused by global warming. Others (Curtsdotter et al., 2019; Jonsson et al., 2018) predicted observed effects of species loss and invasion (Romanuk et al., 2009, 2017; Smith‐Ramesh et al., 2017). ATN theory (Martinez, 2020) has been extended to include detritus (Boit et al., 2012), evolutionary and spatial ecology (Allhoff et al., 2015), ontogenetic niche shifts (Bland et al., 2019; Kuparinen et al., 2016), environmental variability (Kuparinen et al., 2018), and mutualism within pollination networks (Hale et al., 2020).

## 3 STRATEGIES

After deriving the observed and inferred biotic potential and abiotic context of an ecosystem from its metagenome, the challenge involves describing the mechanisms by which its characteristics persist and change through time. In contrast to the remarkable consistency of mechanisms involving molecular identities and interactions among organisms, more generic mechanistic consistency among ecosystems that is apparent in highly conserved patterns in organismal behavior and their interactions (Evans et al., 2013; Martinez, 2020) could be leveraged. For example, organisms' metabolic, growth, and maximum consumption rates typically increase as a three‐quarters power law of their body size (Brown et al., 2004). Additionally, feeding interactions from herbivory through carnivory and parasitism to decomposition that determine carbon and population dynamics are highly conserved both within evolutionary lineages (Davies, 2021; Edger et al., 2015) and among ecosystems (Williams & Martinez, 2008). Mechanisms responsible for this consistency include the trophic hierarchy whereby energy necessarily flows from autotrophs through heterotrophs at higher trophic levels and ultimately to decomposers. That mechanistic constraint plus a species‐level mechanism that constrains generalists to consume organisms adjacent in this hierarchy enables the trophic “niche model” to successfully predict food‐web characteristics (e.g., means and variances of species' trophic levels, generality, vulnerability, connectedness, etc.) within a wide range of terrestrial and aquatic ecosystems (Dunne et al., 2004; Williams & Martinez, 2000, 2008) including half‐billion‐year‐old Cambrian ecosystems (Dunne et al., 2008). Yet, more constraints emerge from the relatively consistent body size ratios between consumers and resources such as predators and their prey (Brose et al., 2019) and parasites and their hosts (Hechinger, 2013). Allometric trophic network (ATN) models (Table 1) integrate these constraints with the identities and abundances of species as inputs into relatively comprehensive models of the structure and dynamics of complex ecosystems. ATN model's output richly describes ecosystems' metaphenomes including how the abundance of multiple species change over time when subjected to environmental change, biodiversity loss, and extraction of ecosystem services (Table 1).

Augmenting this ATN approach (Martinez, 2020) with the greatly increased resolution and precision of network interactions derived from metagenomes may predict metaphenomes as modular, differential‐equation‐based, whole‐cell modeling efforts have predicted phenotypes (Karr et al., 2012). One ATN module may determine network structure from empirical observations assisted by the probabilistic niche model (Williams et al., 2010) and other statistical approaches (Young et al., 2021) to link uncertainty. Another module parameterizes the equations with metabolic and consumption rates again either specifically from empirical observations or from those allometrically derived from the metabolic theory of ecology (Brose et al., 2017; Martinez, 2020; Silva et al., 2022). A third module runs the simulations with a 1‐day time step in contrast to the 1‐second time step of whole‐cell models. Other approaches may mimic physical models of whole cells that simulate the spatial structure and dynamics of each molecule within a cell by doing the same for each organism within an ecosystem (Katz et al., 2011). More plausible may be hybrid approaches where small organisms including microbes are simulated using differential equations (Jansson & Hofmockel, 2018; Weitz et al., 2015) while large organisms are simulated with individual‐based models (DeAngelis, 2018). Rapid advances in automated observation (Dell et al., 2014), large‐scale computing, and eNA‐based analyses will make current limitations (Table 1) much less prohibitive in the near future when modeling whole environmental systems may eventually focus on socio‐ecological sustainability (Davies et al., 2016; Martinez et al., 2012).

## 4 LIMITATIONS

While current limitations are numerous, the most severe are broader than those itemized in Table 1. First among the broad limitations is imagination. Ecology and evolution have long been focused on natural history and “postdiction.” This typically involves extensive observation and documentation compiled early on in field guides, biogeographic maps, and museum collections followed by rigorously constructing and reconstructing explanations of biogeographical patterns, organisms' phylogenies, and experimental results. The quantitatively predicative aspects of these endeavors often fail to explain more than a small fraction of the observed variation. One prominent example concerns biodiversity and ecosystem function. Many years of research were spent merely determining whether their relationship was significantly and meaningfully positive, an issue of obvious and profound importance. More recent efforts typically explain <10% of variation in function within experiments testing its relationships to biodiversity (Cardinale et al., 2012). Similarly, much research attempts to find statistically significant signals of, for example, competition or coevolution. Many researchers are uncertain as to which mechanisms beyond stochasticity to include when making detailed predictions. Discussion of accurate, general, and precise predictions based on, for example, inheritance and consumer‐resource mechanisms, is often like discussing an around‐the‐world trip with a flat earther. It simply makes no sense given the limited world views of contemporary environmental biologists who often describe ecology as a “sick science” (Simberloff, 1981) where “the only law is ‘it depends’” (Lawton, 1995) (sometimes called “context dependence,” Catford et al., 2022).

Such limitations come in no small part from the ongoing exclusion of those outside of an exceedingly narrow demographic from environmental biology which is overwhelmingly dominated by white males. This exclusion of 92% of the world's humans blatantly contradicts the universal domain of science. It also contradicts the central role of ecology and evolution in the profoundly existential problem of sustainability of all species by excluding those from what are the most empirically sustainable cultures such as Aboriginal Australians, Native Americans throughout the Western Hemisphere, and East and South Asians. Instead, ecology and evolution centers the world views from arguably the least sustainable cultures such as those in and emerging from Europe. The intellectual strangle hold necessary to demographically limit participation in environmental biology also severely limits the ability of scientists to imagine and realize innumerable possibilities, most notably here, predicting ecosystem metaphenomes from community metagenomes. Like Europeans trapped in the flat‐world mindset long after others traveled throughout the planet, ecology and evolution is wedded to an overly narrow and highly limited view of scientific exploration. Ending or at least challenging the intellectual blindfolders well‐illustrated by demographic narrowness (Bernard & Cooperdock, 2018) may be one of the most important strategies to meeting many challenges including the one focused on here.

Such narrowness often excludes predicting metaphenotype from metagenotype by asserting that ecosystems are too complex for such ambitions to be realized (Lawton, 1995). For example, many hold the great variability among organisms of the same species and within organisms over time not to mention interactions among organisms prohibits the predictive success achieved by those who study molecules within organisms. This difference between environmental and molecular biology is often attributed to the very low or at least much less relevant variability within molecular species than within organismal species. While this hypothesized consequence of variability may or may not be valid, the purpose of scientists is to generate and test such hypotheses while attempting to discover knowledge that enables successful prediction. Such discovery is unlikely to be achieved unless it is attempted. Attempting to predict metaphenotype from metagenotype would illuminate the extent and limitations of prediction within environmental biology in a much more informed manner than speculations based on thought experiments such as those above that consider organismal and other variability.

Among those not wedded to world views that exclude meeting challenges such as predicting metaphenotype from metagenotype, limitations are still quite evident (Table 1) but not obviously insurmountable 2020. An initial effort in meeting this challenge would include conferences and other collaborations involving a wide range of biologists and other (e.g., information) scientists that generate more agreement about current limitations and new approaches to meeting the challenge. The structured collaborations developed in the genotype‐to‐phenotype grand challenge provide extremely useful lessons for how to, and how not to, organize scientists to pursue a similar challenge at the ecosystem level. For example, norms, standards, and information technology for describing and aggregating organisms and their interactions (Poelen et al., 2014; Simons & Poelen, 2017) need to be better developed as biologists have for biomolecules and their interactions. Computer languages and software platforms better suited integrating large amounts of data and interactions need to be developed so that simulations could be more sophisticated, reliable, and reproducible (Gauzens et al., 2019). Studies that more comprehensively record the long‐term dynamics of ecosystems in the laboratory and field are needed to provide an effective empirical base for developing and testing theory and simulations (Boit et al., 2012). Progress along these lines has been relatively slow. For example, the great effort and success of developing critical information technology such as the gene ontology (Consortium, 2019) vastly outstrips that of ecological ontologies (Michener & Jones, 2012). Leading universities spent hundreds of millions of dollars and hired dozens of professors in the field of systems biology that enabled the scale and success of genotype‐to‐phenotype research. Of course, the clearer and more wealthy beneficiaries of systems biology, most notably the pharmaceutical and medical industries were critical to such massive academic investments. The beneficiaries of increased ecological understanding are much less economically and politically concentrated. Though such understanding could potentially greatly increase the health and sustainability of societies throughout the planet, the disparate individuals and industries that would benefit from such increases along with their limited organization represent yet another profound limitation to achieving the challenge.

## 5 POSSIBILITY AND PROMISE OF PURSUIT

At least since Darwin famously described a “tangled bank” of species “so different from each other, and dependent on each other in so complex a manner, [that] have all been produced by laws acting around us,” scientists have sought to discover how the “integrity of eco‐systems” (Thorpe, 2000) emerges from the complex interactions among organisms in nature. While many biologists focused on how molecules such as DNA determine the structure and function of organisms, environmental biologists focus more on how organisms determine the structure (e.g., diversity) and function (e.g., dynamics) of ecosystems. Both fields focus on highly complex biological systems with many different types of interactions operating on vastly different scales. However, compared to environmental biologists, cell and molecular biologists have more ambitiously and successfully achieved whole system understanding in terms of empirical and mechanistic richness, computational sophistication, and predictive ability. The grand challenge to predict ecosystem metaphenomes from community metagenomes aims to collaboratively leverage the talents and achievements of the latter group for the benefit of the former. While still focusing on nucleic acids, the shift from cell to ecosystem shifts genome analyses from *understanding relationships* among genes (genotype), their interactions (transcription) and their products (proteome), and their products' interactions (metabolome, Fang et al., 2020) to a more straightforward *identification* of organisms and their interactions (Figure 1). Despite this fundamental shift from genes and biochemical species to taxonomic species, much of the conceptual, mathematical, and computational methods are remarkably similar. Focusing on relatively simple labs and natural ecosystems with few species increases the tractability of early stages and provides guidance for later stages. The tractability of such later stages addressing more complex ecosystems is aided by the metagenome's relatively straightforward identification role (as opposed genomes' mechanistic role in generating phenotypes) that helps compensate for the greater variation of ecological networks compared to highly conserved biochemical networks.

Pursuing similar grand challenges in both fields also addresses other key challenges. They include “integration of heterogenous databases, identification of the limits of our knowledge, predicting complex, multi‐network phenotypes, and suggesting future experiments that may lead to new knowledge” (Carrera & Covert, 2015). Perhaps most importantly, both pursue more comprehensive and synthetically predictive understanding of the biological systems they study (Figure 1). Interdisciplinary collaborations that leverage these similarities promise fresh approaches to some of the most difficult environmental problems on the planet.

## AUTHOR CONTRIBUTIONS

**Neo D. Martinez:** Conceptualization (lead); investigation (lead); methodology (lead); project administration (lead); resources (lead); supervision (lead); visualization (lead); writing – original draft (lead); writing – review and editing (lead).

## Data Availability

Data sharing is not applicable to this article as no new data were created or analyzed in this study.
